# A case report of *Klebsiella aerogenes-*caused lumbar spine infection identified by metagenome next-generation sequencing

**DOI:** 10.1186/s12879-022-07583-0

**Published:** 2022-07-15

**Authors:** Huajie Gu, Qingqing Cai, Xiaoyong Dai, Huanhuan Wang, Wenying Xu, Xuejie Cao, Youwen Ye

**Affiliations:** 1 Department of Emergency Intensive Care Unit, Yangpu Hospital, School of Medicine, Tongji University, 450 Tengyue Road, Shanghai, China; 2 Genoxor Medical Science and Technology Inc., Shanghai, China; 3 Department of Radiology, Yangpu Hospital, School of Medicine, Tongji University, Shanghai, China

**Keywords:** Case report, *Klebsiella aerogenes*, Spinal infection, Blood culture, Metagenome next-generation sequencing

## Abstract

**Background:**

The early clinical diagnosis of spinal infections in elderly patients with recessive or atypical symptoms is difficult. *Klebsiella aerogenes* is a common opportunistic bacterium that can infect the respiratory tract, urinary tract, and even the central nervous system. However, whether it can infect the lumbar spine has not been previously described.

**Case presentation:**

In this paper, we report the case of a 69-year-old female patient with osteoporosis who was initially diagnosed with hemolytic anemia. Later, she was diagnosed with *K. aerogenes* infection of the lumbar spine based on imaging combined with blood culture and metagenome next-generation sequencing (mNGS) detection. After precise medication, the lumbar degeneration was improved.

**Conclusions:**

Bacterial infection should therefore be considered in cases of lumbar degenerative disease in middle-aged and elderly patients.

**Supplementary Information:**

The online version contains supplementary material available at 10.1186/s12879-022-07583-0.

## Background

*Klebsiella aerogenes*, formerly known as *Enterobacter aerogenes*, belongs to the family Enterobacteria and is a facultative Gram-negative anaerobe [1]. It is widely distributed in the environment and is found in the human gastrointestinal tract, also being a common opportunistic pathogen in hospitals. When the host immune system is compromised or the intestinal mucosa is damaged, it may cause infection of the respiratory, circulatory, or urogenital system [2]. In recent years, despite increasing reports on the pathogenicity and drug resistance of *Escherichia coli* and *Klebsiella pneumoniae*, there have only been a few reports on *K. aerogenes* [3, 4]. Previous clinical reports on this bacterium were mainly of respiratory tract, gastrointestinal tract, urinary tract, and blood infections. Compared with other Enterobacteriaceae species, *K. aerogenes* is more likely to cause septic shock or even death in patients[5, 6]. To date, lumbar infections caused by *K. aerogenes* have not been reported. Herein, we report the first case of a patient with a lumbar *K. aerogenes* infection.

## Case presentation

A 69-year-old woman was hospitalized on July 30, 2020, with recurrent fever without any cause for two weeks and continuous lower back pain for three days. Three months prior, she presented with chest discomfort and fatigue without an obvious cause, occasional chest pain after activity, and frequent urination at night. She did not have paroxysmal nocturnal dyspnea, fever, cough, expectoration, urinary pain, nausea, vomiting, abdominal pain, abdominal distension, or diarrhea. Physical examination revealed high inflammatory indices, including a white blood cell (WBC) count of 21.7 × 10^9^ cells/L, a C-reactive protein (CRP) level of 12.46 mg/L, and procalcitonin (PCT) level of 0.19 ng/mL. She was treated for infection and anemia, as laboratory tests revealed an erythrocyte count of 1.00 × 10^12^/L, a hematocrit of 14.0%, a hemoglobin concentration of 43 g/L, a mean corpuscular volume (MCV) of 140.0 fL, a mean corpuscular hemoglobin (MCH) level at 43.0 Pg, and a MCH concentration of 307 g/L (Table 1). The symptoms did not improve, and the patient remained at our hospital for treatment.

**Table 1:** The results of the laboratory test in the patient

Laboratory tests	
WBC (×10^9^/L)	21.7
CRP (mg/L)	12.46
PCT (ng/mL)	0.19
Hemoglobin concentration (g/L)	43
MCV (fL)	140.0
MCH (Pg)	43.0
MCH concentration (g/L)	307

**Fig. 1:**
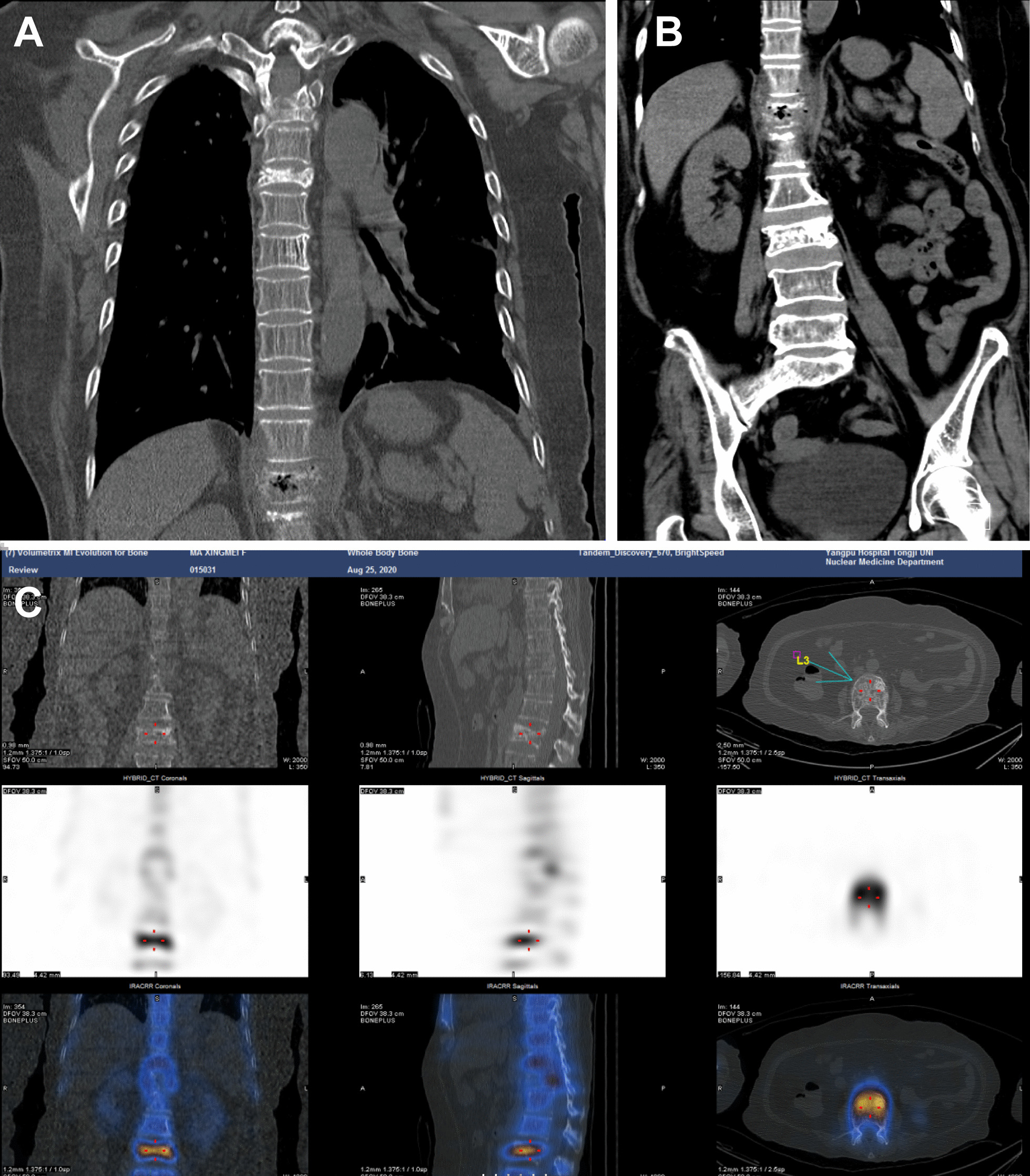
Thoracolumbar imaging findings. **A** Chest CT on August 18, 2020. **B** Abdominal CT on August 18, 2020. **C** Radionuclide scan on August 25, 2020

The chief complaint at admission: In 2006, the patient underwent radical mastectomy and chemotherapy after surgery, with a 7-year history of hypertension and continuous lower back pain for three days. Through physical examination, it was found that the patient had back tenderness and paravertebral percussion pain, leading to the diagnosis of thoracic degenerative changes, particularly compression changes at the fifth and the twelfth thoracic as well as the third lumbar vertebra. Body temperature fluctuated between 38.6 and 38.8 °C, and the patient was treated with ceftriaxone and ceftazidime. On August 18, she was hospitalized again due to recurrent fever for 2 weeks and severe diarrhea for 1 day. Chest computed tomography (CT) revealed the absence of soft tissue shadows in the right breast and right armpit, increased texture in both lungs, a cord-like shadow in both lungs, a small amount of left pleural effusion, and bilateral pleural thickening (Fig. 1A).

Abdominal CT showed a slightly enlarged spleen, local pneumatosis expansion in the proximal colon ascendens, and no other visible abnormalities (Fig. 1B). Blood cultures were positive for *K. aerogenes* within 15 h (Table 2), and the result of urine cultures was negative, while stool cultures were positive for *Candida albicans* and negative for *Enterobacter* sp. and *Vibrio casei*. Based on drug sensitivity testing and the patient’s condition, meropenem and diflucan (fluconazole resistance) were administered against infection. Detailed information on the drug sensitivity testing was provided in the Additional file 1: Online Technical Appendix. After 4 days, body temperature was normal, and the patient was treated with sulperazon.

**Table 2:** The results of blood cultures within 15 h

Antibacterial agents	Break point	Results	Interpretation
The results of blood cultures: positive for *Klebsiella aerogenes*			
MIC method (unit: mg/mL)			
Piperacillin-Tazobactam	≤ 16 ≥128	≤ 4	Sensitivity
Ceftazidime	≤ 4 ≥16	≤ 0.12	Sensitivity
Cefoperazone-Sulbactam	≤ 16 ≥64	≤ 8	Sensitivity
Cefepime	≤ 2 ≥16	≤ 0.12	Sensitivity
Aztreonam	≤ 4 ≥16	≤ 1	Sensitivity
Imipenem	≤ 1 ≥4	2	Intermediate
Meropenem	≤ 1 ≥4	≤ 0.25	Sensitivity
Amikacin	≤ 16 ≥64	≤ 2	Sensitivity
Tobramycin	≤ 4 ≥16	≤ 1	Sensitivity
Ciprofloxacin	≤ 0.25 ≥1	≤ 0.25	Sensitivity
Levofloxacin	≤ 0.5 ≥2	≤ 0.12	Sensitivity
Doxycycline	≤ 4 ≥16	1	Sensitivity
Minocyline	≤ 4 ≥16	4	Sensitivity
Tigecycline	≤ 2 ≥8	1	Sensitivity
Colistin	≤ 2 ≥4	≤ 0.5	Sensitivity
Bactrim	≤ 40 ≥80	≤ 20	Sensitivity
Results of blood cultures: no anaerobic bacterium was found			
K-B method (unit: mm)			
Cefazolin	≥ 23 ≤19	6	Resistance
Gentamicin	≥ 15 ≤12	23	Sensitivity
Ampicillin	≥ 17 ≤13	6	Resistance
Ampicillin-Sulbactam	≥ 15 ≤11	6	Resistance
Cefuroxime	≥ 18 ≤14	20	Sensitivity
Cefuroxime axetil	≥ 23 ≤14	20	Intermediate
Cefoxitin	≥ 18 ≤14	6	Resistance
Meropenem	≥ 23 ≤19	25	Sensitivity
Imipenem	≥ 23 ≤19	21	Intermediate
Cefotaxime	≥ 23 ≤19	28	Sensitivity
Amoxicillin-clavulanic acid	≥ 18 ≤13	6	Resistance
Ceftazidime-avibactam	≥ 21 ≤20	25	Sensitivity
Results of smear: the gram-negative bacteria were found			

On August 25, the patient, who had a history of breast cancer, experienced chest discomfort. A bone scan with the whole-body bone scintigraphy was performed, confirming the absence of bone metastases, while also revealing cuneiform changes at the third, fifth, twelfth thoracic vertebrae, and the third lumbar vertebra, indicative of degenerative changes in the thoracolumbar spine (Fig. 1C). During follow-up from anemia treatment, the patient had a normal hemoglobin level and was administered oral prednisone at a dose of 10 mg bid. The patient’s D-dimer levels remained high, and an improved pulmonary artery CT angiography (CTA) suggested that the small branch was embolized in the lower lobe of both lungs. The patient was treated with low-weight molecular heparin and switched to oral anticoagulants (rivaroxaban) when under stable condition.

On August 27, the patient returned to a normal body temperature, and antibiotics were discontinued. On August 31, the patient had increased PCT level, so she was once again treated with sulperazon against infection until she was discharged on September 12. There was no significant change in the blood routine and PCT indices during this period. She experienced a fever again without an obvious cause one day after discharge. Upon admission, the highest body temperature was 39.5 °C, accompanied by aversion to cold, chills, and fatigue. Physical examination revealed a CRP level of 88.89 mg/L, a WBC count of 14.4 × 10^9^ cells/L, a body temperature of 40.4 °C, and shock. *K. aerogenes* infection was detected through blood cultures within 12 h, and the patient was subsequently treated with meropenem against infection.

**Fig. 2:**
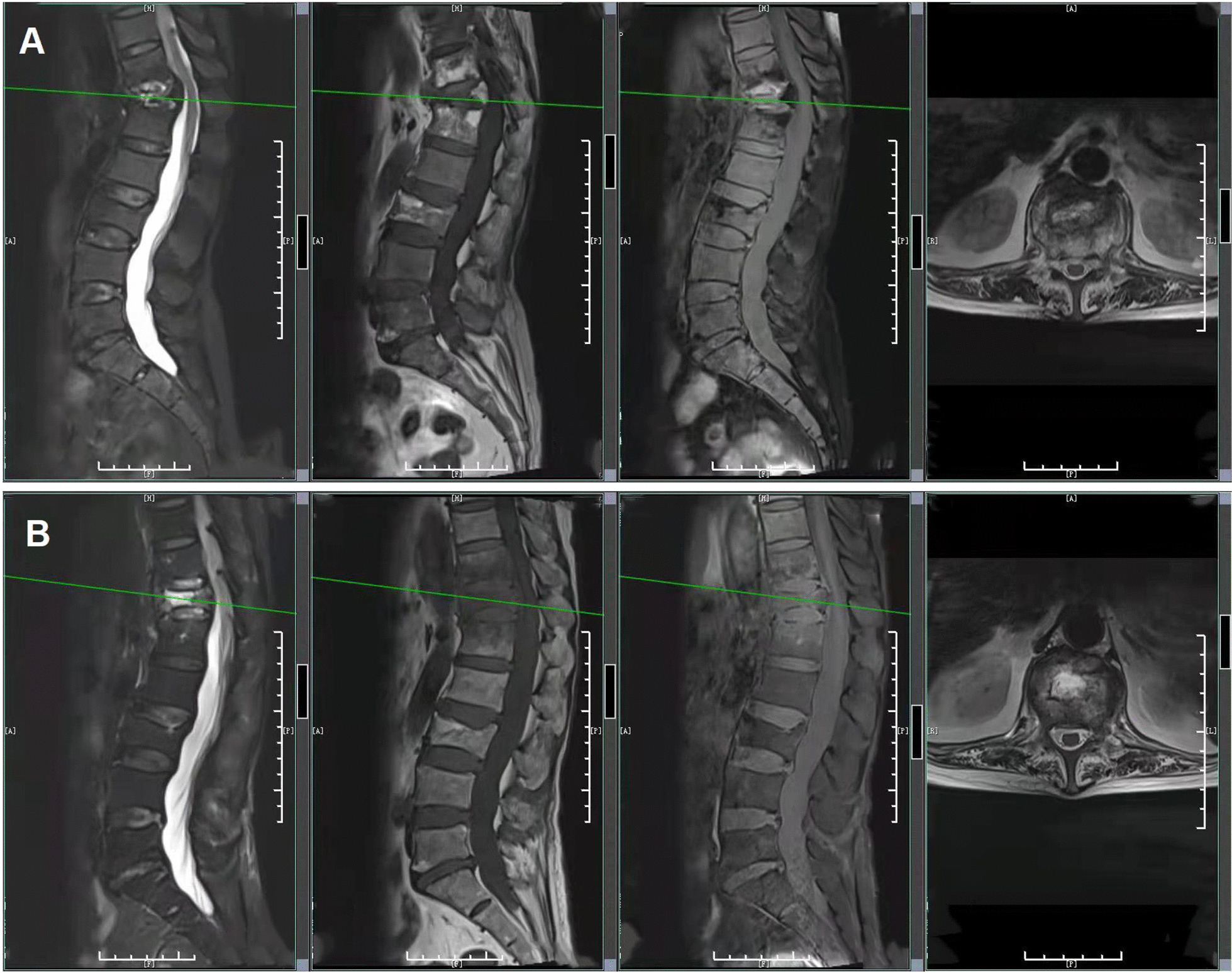
**Results of the MRI in lumbar and thoracic vertebrae. A** MRI of the lumbar and thoracic vertebrae on September 17, 2020. **B** MRI of the lumbar and thoracic vertebrae on October 10, 2020

A CT scan of the thoracoabdominal region showed significant changes in the twelfth thoracic vertebra. Abdominal CT revealed vertebral compression change of the third lumbar vertebrae. MRI of the lumbar spine showed bone destruction from the eleventh thoracic vertebrae to the first lumbar vertebrae, protrusion of the intervertebral disc at the fifth lumbar vertebra to the first sacral vertebra, and degenerative changes in the lumbar spine (Fig. 2A ). Therefore, we considered that the patient had a vertebral infection. The patient experienced recurrent vague back pain, which she tolerated over half a year. Considering bone destruction, the expert consultation from another hospital led to the administration of cefepime combined with levofloxacin for the treatment of infection. One day later, the patient once again had a fever, and the axillary temperature was 38.5 °C, with a CRP level of 47.9 mg/L and a WBC count of 14.6 × 10^9^ cells/L. The antibiotic was changed to meropenem combined with moxifloxacin, and the following two times of blood cultures were negative.

On October 10, MRI of the lumbar spine revealed bone destruction from the eleventh thoracic vertebra to the first lumbar vertebrae. The thoracic MRI indicated mild deviation with signal changes of the eleventh thoracic vertebrae and the first lumbar vertebrae as well as vertebral compression with signal abnormalities of the twelfth thoracic vertebrae (Fig. 2B). From October 16 to 29, blood cultures were negative twice in a row, but *K. aerogenes* was detected via metagenomic next-generation sequencing (mNGS) on the 21st (Table 3). The specific methods of mNGS were described in the Additional file 1: Online Technical Appendix. Cefepime and moxifloxacin were administered continuously. The patient’s body temperature and inflammatory indexes tended to be normal during the treatment. After discharge, the patient continued oral administration of levofloxacin as well as doxycycline for infection treatment and was instructed to rest in bed to prevent falls, avoid stress on the waist, and appropriately move the lower limbs to avoid thrombosis. During the follow-up period, routine blood tests, inflammatory indexes, and body temperature were normal.

**Table 3:** The results of metagenomic next-generation sequencing

Types	Species			Genus	
	Name	Reads	Relative abundance	Name	Reads
Gram negative bacteria	*Klebsiella aerogenes*	200	3.87%	*Klebsiella*	215

## Discussion and conclusions

The present work describes case of a lumbar spine infection caused by *K. aerogenes*. The treatment process was complex, mainly due to the older age of the patient, the insignificant symptoms of lumbar infection, and no previous experience with spinal *K. aerogenes* infections. After repeated CT reexamination and communication with the patient about her physical symptoms, the therapeutic results were satisfactory. Based on our observations, we suggest that the possibility of spinal bacterial infection should be fully considered, and rare spinal pathogens, such as *K. aerogenes*, should be included in the diagnostic scope when treating elderly patients who develop lumbar degenerative diseases.

*K. aerogenes*, which has a thick capsule, belongs to the genus *Klebsiella* of Enterobacteriaceae. It is a Gram-negative facultative anaerobe that is widely distributed in the natural environment and animal intestines [7]. Like other Enterobacteriaceae pathogens, *Klebsiella* virulence and drug resistance are complex and influenced by multiple factors. The produced virulence factors and the drug resistance genes can vary depending on the site of infection. Adhesins produced by *Klebsiella* facilitate its entry into host cells, with capsular polysaccharides and lipopolysaccharides on the cell surface helping the bacteria to escape from the phagocytosis, while toxins or other extracellular components cause mucosal damage and spread through the circulation. In recent years, due to the excessive use of antibiotics, an increasing number of *Klebsiella* species have developed multidrug resistance [8]. As reported, carbapenem-resistant *K. aerogenes* isolates were previously found in clinical studies in China [9, 10]. In our present study, drug sensitivity test results indicated that *K. aerogenes* isolates were non-resistant bacteria to carbapenems, and were especially sensitive in meropenem, which was given as anti-infection treatment. In addition, the isolated *K. aerogenes* was intermediate in imipenem. Nevertheless, they were found to be resistant to penicillins, such as ampicillin and amoxicillin. Generally, *Klebsiella*-related infections develop rapidly, causing multiple organ failure or even death, and drug-resistant strains easily arise. Thus, effective antibiotics should be selected as early as possible, and the dose as well as course of treatment should be determined to minimize the occurrence of side effects.

Due to the lack of specific symptoms and signs, the early diagnosis of spinal infections is relatively difficult. Clinicians have to differentiate it based on characteristics of the disease, clinical symptoms, and signs, combined with X-ray, CT, MRI, and other imaging findings. Spinal infection is mostly subacute or chronic, and generally occurs in patients with weakened immune function or spinal surgery. In recent years, spinal infections have been reported to be caused by dental problems in adult patients with normal immunity [11]. Pain at the lesion site is the initial symptom, which may be accompanied by an elevated body temperature. Pathogens in patients with spinal infectious diseases are usually identified by blood culture. The common pathogens are *Staphylococcus aureus*, *Streptococcus* sp., *Brucella* sp., and *Mycobacterium tuberculosis* [12–14]. At present, there are no published case reports of *K. aerogenes* spinal infection. The patient in this study, an elderly female, was diagnosed with K. *aerogenes* infection of the lumbar spine based on imaging combined with blood culture and mNGS detection. After diagnosis and precise medication, lumbar degeneration was improved, and the patient was satisfied with the treatment. The present case highlights the possibility of spinal bacterial infection when lumbar degeneration is observed in middle-aged and elderly patients.

## Supplementary information

**Additional file 1: Technical Appendix.**

## Data Availability

The datasets used and/or analyzed during the current study are available from the corresponding author on reasonable request.

## Abbreviations

MNGS: Metagenome next-generation sequencing
WBC: White blood cell
CRP: C-reactive protein
PCT: Procalcitonin
CT: computed tomography
CTA: Computed tomography angiography
MCV: mean corpuscular volume
MCH: Mean corpuscular hemoglobin
