# Supplementary material for: A case report of Klebsiella aerogenes-caused lumbar spine infection identified by metagenome next-generation sequencing
Source: BMC Infect Dis. 2022 Jul 15;22:616. doi: 10.1186/s12879-022-07583-0 (PMC9288078; doi:10.1186/s12879-022-07583-0)
Supplement: Supplementary file 1 — Additional file 1: Technical Appendix. [file 12879_2022_7583_MOESM1_ESM.docx]

**Technical appendix**

**Drug susceptibility test**

Method used for susceptibility testing was varied among different pathogens. Broth microdilution and Kirby-Bauer (KB) methods were conducted for the bacteria test, including the *Klebsiella aerogenes*. The fungi were tested by using the broth microdilution method. The minimal inhibitory concentration (MIC) was obtained with broth microdilution performed according to CLSI guideline (28th Edition, 2018) with each isolate tested in duplicate; a third replicate was necessary if there was disagreement between the first two broth microdilution results. For Kirby-Bauer method, plates were incubated at 35 °C and read after 16–20 h incubation, disk containing different contents of drugs were used for disk diffusion test, k diffusion tests were performed in triplicate in parallel with broth microdilution, the mean value of the three inhibition zone diameter is used for statistical analysis. Escherichia coli ATCC 25922 and Escherichia coli ATCC 32518 were used for quality control.

**Metagenomic next-generation sequencing (mNGS)**

Blood samples taken from the patients were drawn into 10-mL Vacuumm blood collection tubes (Kang Jian, CHN), and centrifuged to remove cells for reduction of the host-background nucleic acid. Subsequently, cf DNA was extracted using Magnetic Serum/ Plasma DNA Maxi Kit (TIANGEN, CHN) following the manufacturer’s instructions. DNA extraction yield was quantified using a QuantiT dsDNA HS Assay Kit and Qubit 3.0 Fluorometer (Thermo Scientific, USA). Enzymatic shearing was employed for the fragmentation (~200 bp) of DNA molecules and the libraries were then constructed using the Nextera XT DNA Library Preparation Kit (Illumina, USA). The quality of the libraries was assessed by a 2100 Bioanalyzer using the High Sensitivity DNA Assay (Agilent Technologies, USA). Metagenome shotgun sequencing in a single-end 75-bp mode was performed using the NextSeq 500/550 High Output Kit (92 cycles) on an Illumina NextSeq 550 Dx sequencer. The samples as the No-Template Control (NTC) were sequenced simultaneously to assess contaminations during the wet-lab experiments. The sequence data reported in this study was archived in the NCBI's Sequence Read Archive (SRA) with the accession number SRR18550723.

**Bioinformatics analysis of species-level abundance profiling**

Raw sequencing data were first subjected to a quality control process for trimming adapter sequences; removing low-quality tails and reads by Trimmomatic v0.36. Next, the reads mapping to the human reference genome GRCh37 were excluded using the short-read alignment tool Bowtie v2.2.6. Read duplication was then performed using in-house scripts. Taxonomic classification of microbial reads was conducted using Kraken v2.0.9-beta and a custom k-mer database that was constructed using 51,543 genomes of ~27,000 species from the NCBI assembly databases.
